# Supplementary material for: Spiro-Oxindole Skeleton Compounds Are Efficient Inhibitors for Indoleamine 2,3-Dioxygenase 1: An Attractive Target for Tumor Immunotherapy
Source: Int J Mol Sci. 2022 Apr 23;23(9):4668. doi: 10.3390/ijms23094668 (PMC9104902; doi:10.3390/ijms23094668)
Supplement: Supplementary file 1 [file ijms-23-04668-s001.zip › ijms-1701053-supplementary.pdf]

Supplementary Information

Spiro-Oxindole Skeleton Compounds Are Efficient Inhibitors for Indoleamine 2,3-Dioxygenase 1: An Attractive Target for Tumor Immunotherapy

Daojing Yan [1], Jiakun Xu [2], Xiang Wang [1], Jiaxing Zhang [3], Gang Zhao [3], Yingwu Lin [4,*] and Xiangshi Tan [1,*]

[1] Department of Chemistry, Fudan University, 2005 Songhu Road, Shanghai 200433, China;
djyan16@fudan.edu.cn (D.Y.); 17110220109@fudan.edu.cn (X.W.)

[2] Key Laboratory of Sustainable Development of Polar Fisheries, Ministry of Agriculture and Rural Affairs, Yellow Sea Fisheries Research Institute, Chinese Academy of Fishery Sciences, Laboratory for Marine Drugs and Byproducts of Pilot National Laboratory for Marine Science and Technology, Qingdao 266071, China; xujk@ysfri.ac.cn

[3] Key Laboratory of Synthetic Chemistry of Natural Substances, Shanghai Institute of Organic Chemistry, Chinese Academy of Sciences, 345 Lingling Road, Shanghai 200032, China; zhangjiaxing@chemchina.com (J.Z.); zhaog@mail.sioc.ac.cn (G.Z.)

[4] School of Chemistry and Chemical Engineering, University of South China, Hengyang 421001, China

[*] Correspondence: ywlin@usc.edu.cn (Y.L.); xstan@fudan.edu.cn (X.T.)

Contents

1.1 General information

1.2 Procedure for the Synthesis of Spiro[pyrrolidin-3,3'-Oxindoles] Compound 2

1.3 Procedure for the Synthesis of Spiro[pyrrolidin-3,3'-Oxindoles] Compound 3

1.4 NMR & Mass spectra of compound 2 and 3

1.5 Molecular docking results of inhibitor-IDO1 complex

1.1 General information

The mass spectra were acquired using a Q-Exactive™ Focus Hybrid Quadrupole-Orbitrap Mass Spectrometer (Thermo Fisher Scientific Inc.) equipped with a Dionex Ultimate 3000 HPLC system (Thermo Fisher). ^{1}H NMR spectra were recorded on a Bruker (400 MHz). All chemical shifts (δ) were given in ppm. Data were reported as follows: chemical shift, integration, multiplicity (s = single, d = doublet, dd = double doublet, m = multiplet) and coupling constants (Hz). ^{13}C NMR spectra were recorded on a DPX-400 (400 MHz).

The structural characterization of compounds **1** and **4** were shown in the article published by Lab of Prof. Zhao [1].

1.2 Procedure for the Synthesis of Spiro[pyrrolidin-3,3'-Oxindoles] Compound 2 [1,2]

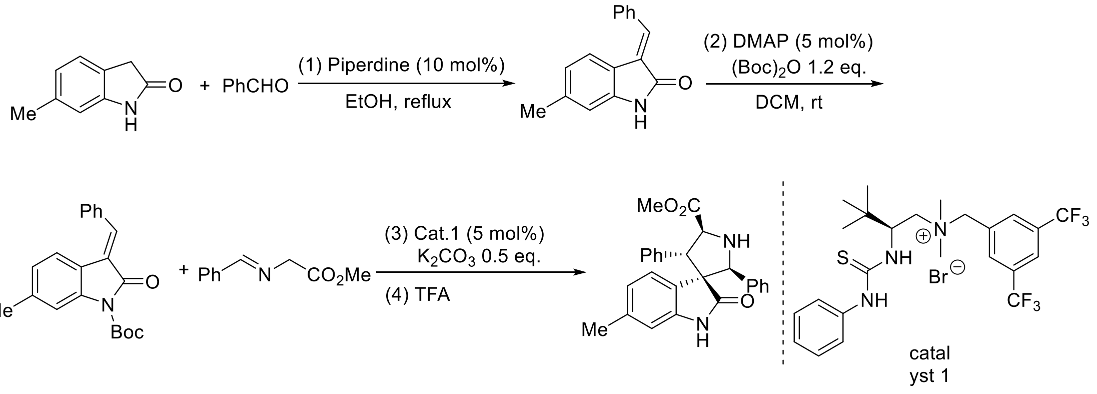

A dry tube was charged with 6-methylindolin-2-one (206.0 mg, 1.4 mmol), sealed with a rubber stopper, evacuated and backfilled with nitrogen for three times before the addition of benzaldehyde (177.8 mg, 1.68 mmol), piperdine (11.9 mg, 0.14 mmol) and

EtOH (3 mL) via syringes. The mixture was refluxing for 8 hours, the reaction was cooled to room temperature. The organic layer was concentrated, and the crude product was purified by flash filter. The solid was dissolved by CH_2Cl_2 (3 mL), then DMAP (8.5 mg, 0.07 mmol) and $(Boc)_2O$ (366.8 mg, 1.68 mmol) were added. After stirring for 1 hour, the reaction was quenched by addition of 25 mL of cold water. The organic layer was then washed with 25 mL cold water, 25 mL brine. The organic layer was dried over Na_2SO_4, filtered, and concentrated. The crude product was added with 0.3 mL of DCM and 3 mL of petroleum ether, and stirred at room temperature for 10 hours to obtain a solid-liquid mixture, which was then filtered to obtain the intermediate product.

A mixture of K_2CO_3 (30.4 mg, 0.22 mmol), catalyst **cat.1** (12.9 mg, 0.022 mmol), *tert*-butyl (*E*)-3-benzylidene-6-methyl-2-oxoindoline-1-carboxylate (147.6 mg, 0.44 mmol), and in Et_2O (2.0 mL) was stirred at 0 °C. To the mixture was added the methyl (*E*)-2-(benzylideneamino)acetate (117.0 mg, 0.66 mmol), the reaction mixture was stirred at same temperature until the reaction was completed (the reaction time was monitored by TLC). The resultant solution was purified through flash column chromatography on silica gel (eluent: petroleum ether: ethyl acetate = 5:1-3:1) the intermediate products. Next, trifluoroacetic acid (4 mL) was added to a mixture of intermediate products and CH_2Cl_2 (4 mL). After stirring for 6 h at room temperature, the reaction mixture was concentrated under reduced pressure and neutralized with $NaHCO_3$ aq. The organic layer was extracted with CH_2Cl_2, the collected organic layer was dried over anhydrous Na_2SO_4. Concentrated in vacuum and silica gel column chromatography (hexane/ethyl acetate = 2:1) give the pure Compounds **2** (37.0 mg, yield: 90 %, HPLC purity: 98%).

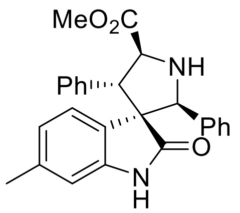

Methyl (2'S,3R,4'S,5'S)-6-methyl-2-oxo-2',4'-diphenylspiro[indoline-3,3'-pyrrolidine]-5'-carboxylate **2**

Compounds 2: 163.3 mg, yield: 90 %, ee: 95 %, light yellow solid; ^{1}H NMR (400

MHz, $CDCl_3$) δ 7.35 – 7.27 (m, 4H), 7.21 (d, J = 6.4 Hz, 2H), 7.17 – 7.07 (m, 4H), 6.96 (d, J = 7.3 Hz, 2H), 6.81 (d, J = 7.7 Hz, 1H), 6.44 (d, J = 7.8 Hz, 1H), 5.80 (s, 1H), 4.70 (s, 1H), 4.59 (d, J = 4.9 Hz, 1H), 4.16 (d, J = 4.9 Hz, 1H), 3.83 (s, 3H), 2.03 (s, 3H). ^{13}C NMR (101 MHz, $CDCl_3$) δ 179.3, 173.2, 139.2, 138.3, 135.7, 131.1, 129.0, 128.4, 128.1, 128.0, 127.9, 127.6, 127.4, 126.3, 126.1, 108.7, 72.4, 66.1, 64.0, 57.3, 52.5, 21.0. ESI: m/z calculated for $(C_{26}H_{24}N_2O_3)H^+$: 413.18, found: 413.18.

1.3 Procedure for the Synthesis of Spiro[pyrrolidin-3,3'-Oxindoles] Compound 3 [1,2]

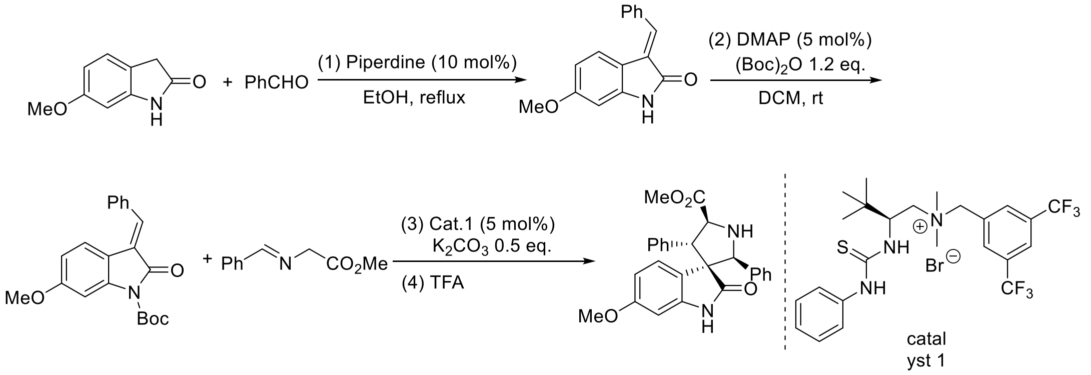

A dry tube was charged with 6-methoxyindolin-2-one (212.1 mg, 1.3 mmol), sealed with a rubber stopper, evacuated and backfilled with nitrogen for three times before the addition of benzaldehyde (165.5 mg, 1.56 mmol), piperdine (11.1 mg, 0.13 mmol) and EtOH (3 mL) via syringes. The mixture was refluxing for 8 hours, the reaction was cooled to room temperature. The organic layer was concentrated, and the crude product was purified by flash filter. The solid was dissolved by CH_2Cl_2 (3 mL), then DMAP (8.5 mg, 0.07 mmol) and $(Boc)_2O$ (340.5 mg, 1.56 mmol) were added. After stirring for 1 hour, the reaction was quenched by addition of 25 mL of cold water. The organic layer was then washed with 25 mL cold water, 25 mL brine. The organic layer was dried over Na_2SO_4, filtered, and concentrated. The crude product was added with 0.3 mL of DCM and 3 mL of petroleum ether, and stirred at room temperature for 10 hours to obtain a solid-liquid mixture, which was then filtered to obtain the intermediate product.

A mixture of K_2CO_3 (24.8 mg, 0.18 mmol), catalyst **cat.1** (10.5 mg, 0.018 mmol),

tert-butyl (*E*)-3-benzylidene-6-methoxy-2-oxoindoline-1-carboxylate (126.5 mg, 0.36 mmol), and in Et_2O (2.0 mL) was stirred at 0 °C. To the mixture was added the methyl (*E*)-2-(benzylideneamino)acetate (95.7 mg, 0.54 mmol), the reaction mixture was stirred at same temperature until the reaction was completed (the reaction time was monitored by TLC). The resultant solution was purified through flash column chromatography on silica gel (eluent: petroleum ether: ethyl acetate = 5:1-3:1) the intermediate products. Next, trifluoroacetic acid (4 mL) was added to a mixture of intermediate products and CH_2Cl_2 (4 mL). After stirring for 6 h at room temperature, the reaction mixture was concentrated under reduced pressure and neutralized with $NaHCO_3$ aq. The organic layer was extracted with CH_2Cl_2, the collected organic layer was dried over anhydrous Na_2SO_4. Concentrated in vacuum and silica gel column chromatography (hexane/ethyl acetate = 2:1) give the pure Compounds **3** (141.9 mg, yield: 92 %, HPLC purity: 98%).

Methyl (2'S,3R,4'S,5'S)-6-methoxy-2-oxo-2',4'-diphenylspiro[indoline-3,3'-pyrrolidine]-5'-carboxylate

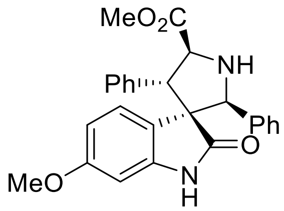

Compound 3: 141.9 mg, yield: 92 %, ee: 95 %, light yellow solid; ^{1}H NMR (400 MHz, $CDCl_3$) δ 7.35 – 7.27 (m, 4H), 7.21 (d, J = 6.4 Hz, 2H), 7.17 – 7.07 (m, 4H), 6.96 (d, J = 7.3 Hz, 2H), 6.81 (d, J = 7.7 Hz, 1H), 6.44 (d, J = 7.8 Hz, 1H), 5.80 (s, 1H), 4.70 (s, 1H), 4.59 (d, J = 4.9 Hz, 1H), 4.16 (d, J = 4.9 Hz, 1H), 3.83 (s, 3H), 2.03 (s, 3H). ^{13}C NMR (101 MHz, $CDCl_3$) δ 179.3, 173.2, 139.2, 138.3, 135.7, 131.1, 129.0, 128.4, 128.1, 128.0, 127.9, 127.6, 127.4, 126.3, 126.1, 108.7, 72.4, 66.1, 64.0, 57.3, 52.5, 21.0. ESI: m/z calculated for $(C_{26}H_{24}N_2O_4)H^+$: 429.18, found: 429.18

1.4 NMR & Mass spectra of compound 2 and 3

Compound 2: ^{1}H-NMR, ^{13}C-NMR, Mass spectra

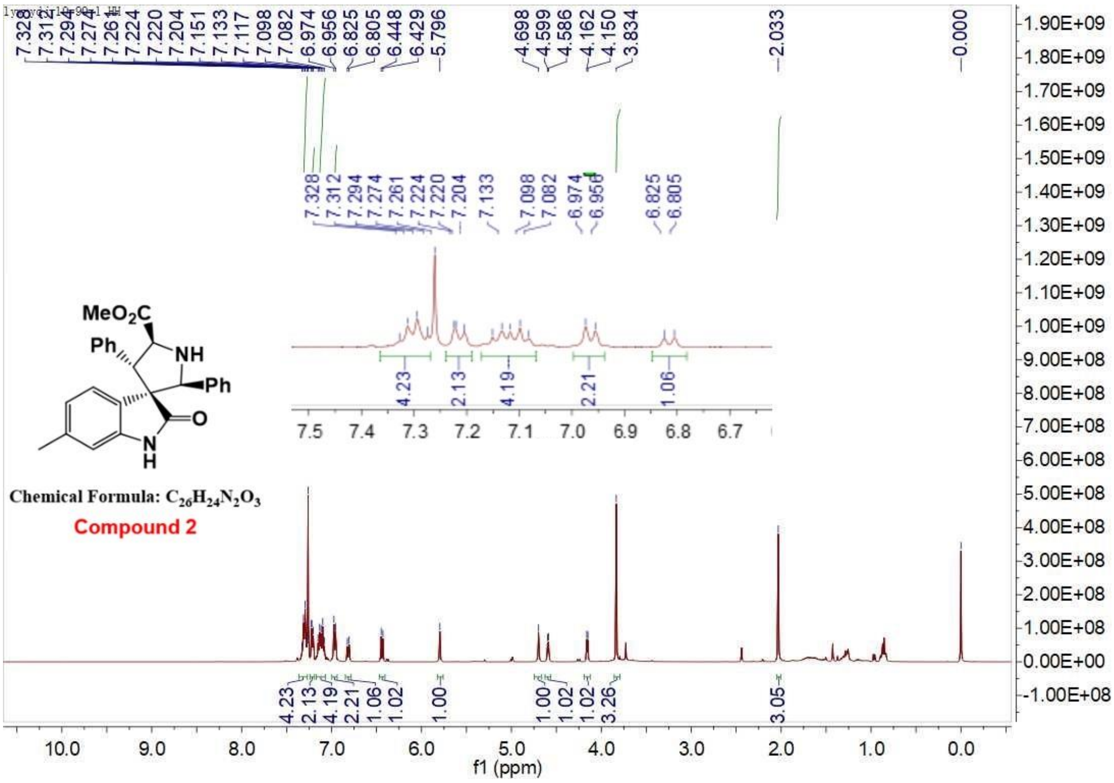

Figure S1. ^{1}H-NMR of **Compound 2**.

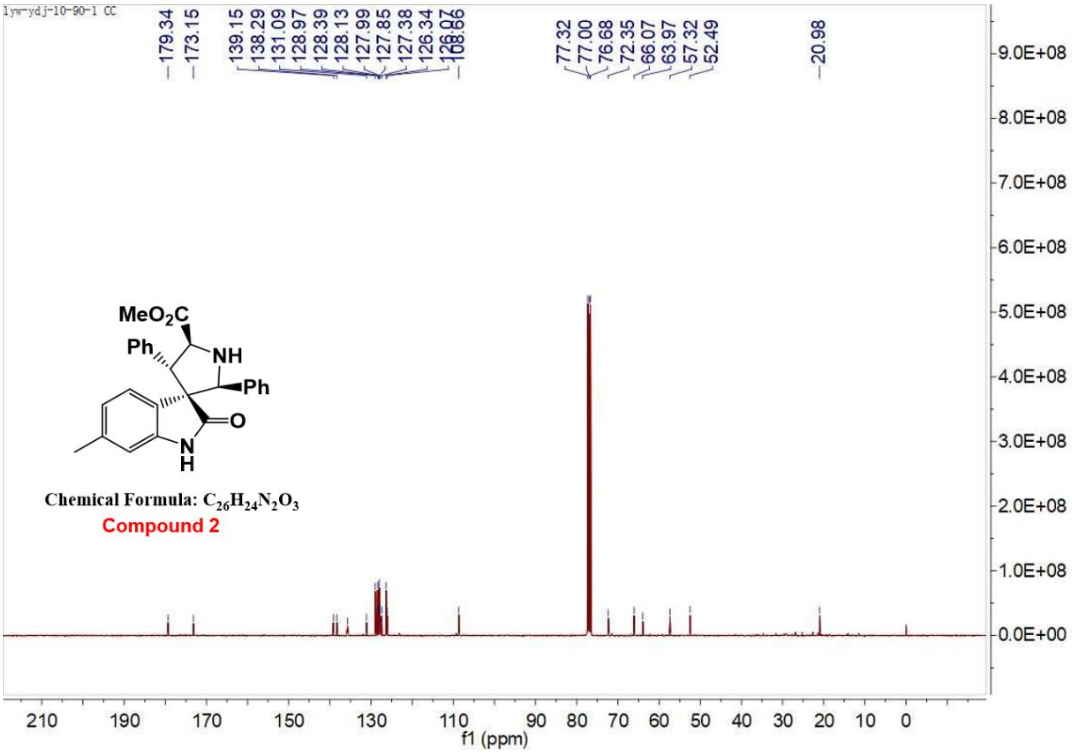

Figure S2. ^{13}C-NMR of **Compound 2**.

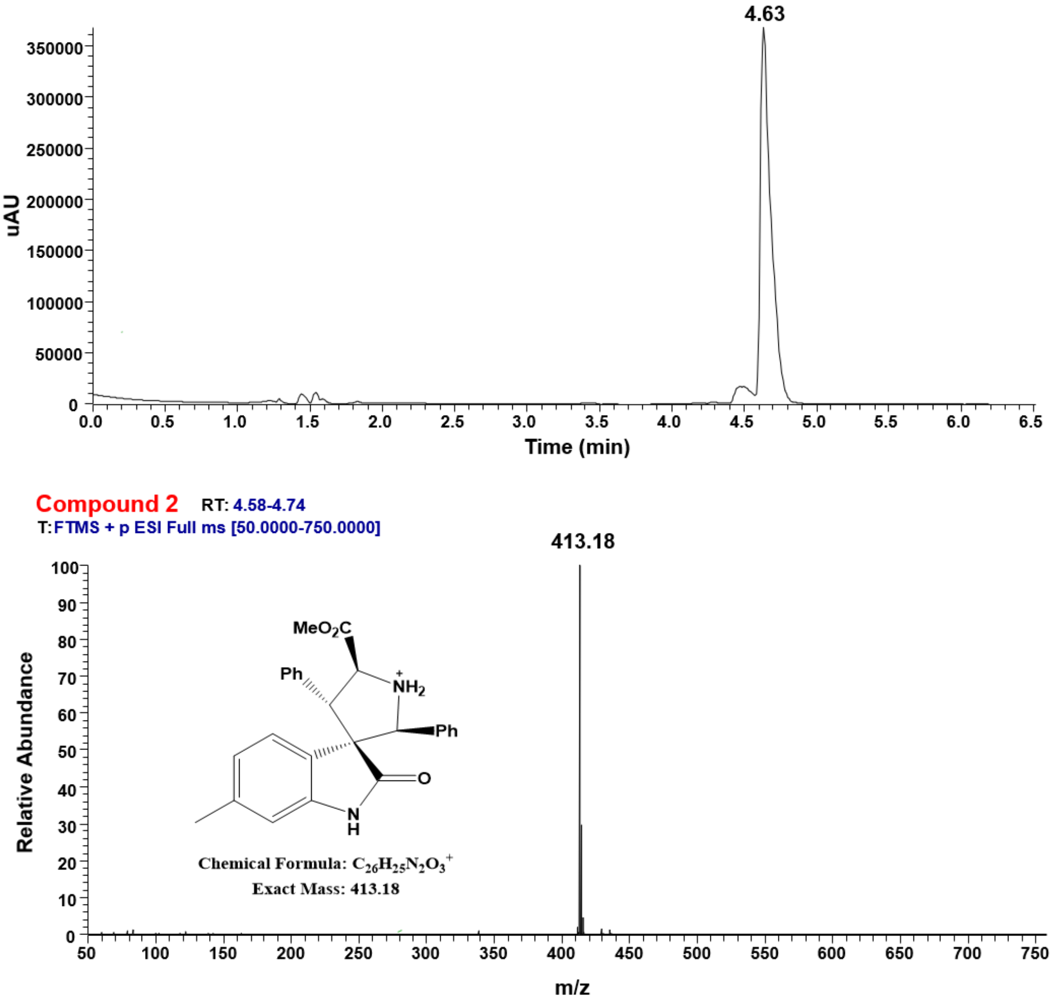

Figure S3. Mass spectra of **Compound 2**.

Compound 3: ^{1}H-NMR, ^{13}C-NMR, Mass spectra

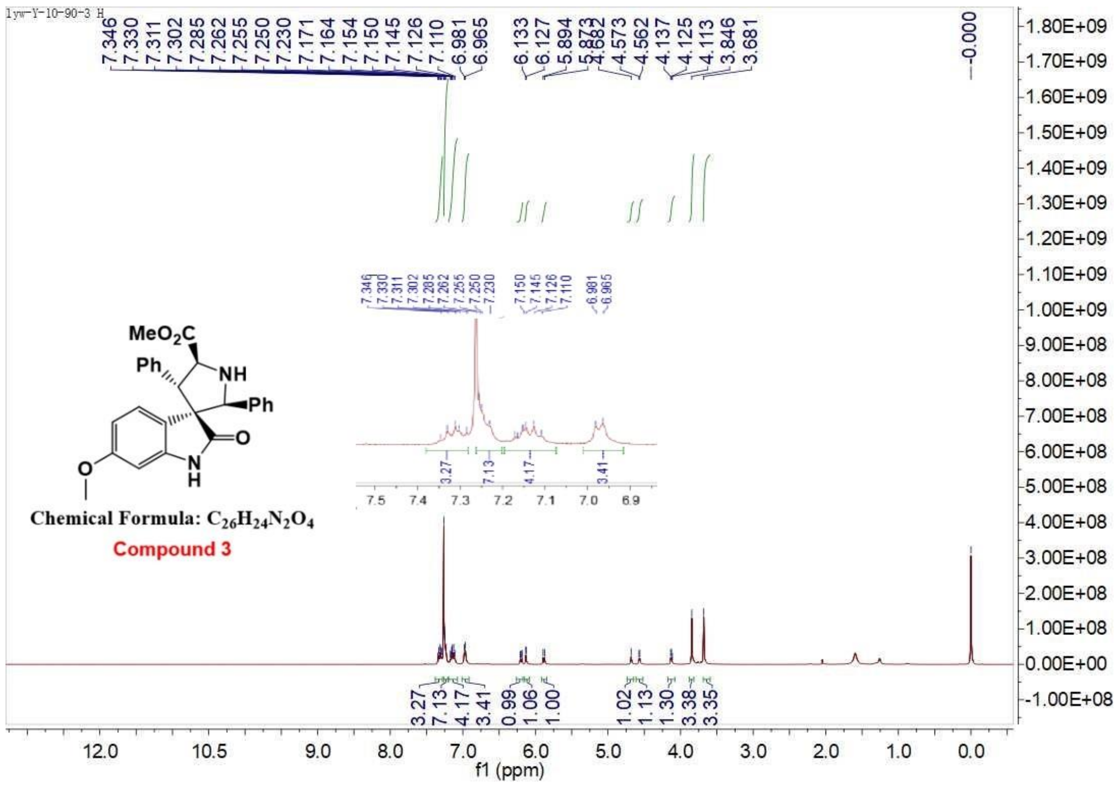

Figure S4. ^{1}H-NMR of **Compound 3**.

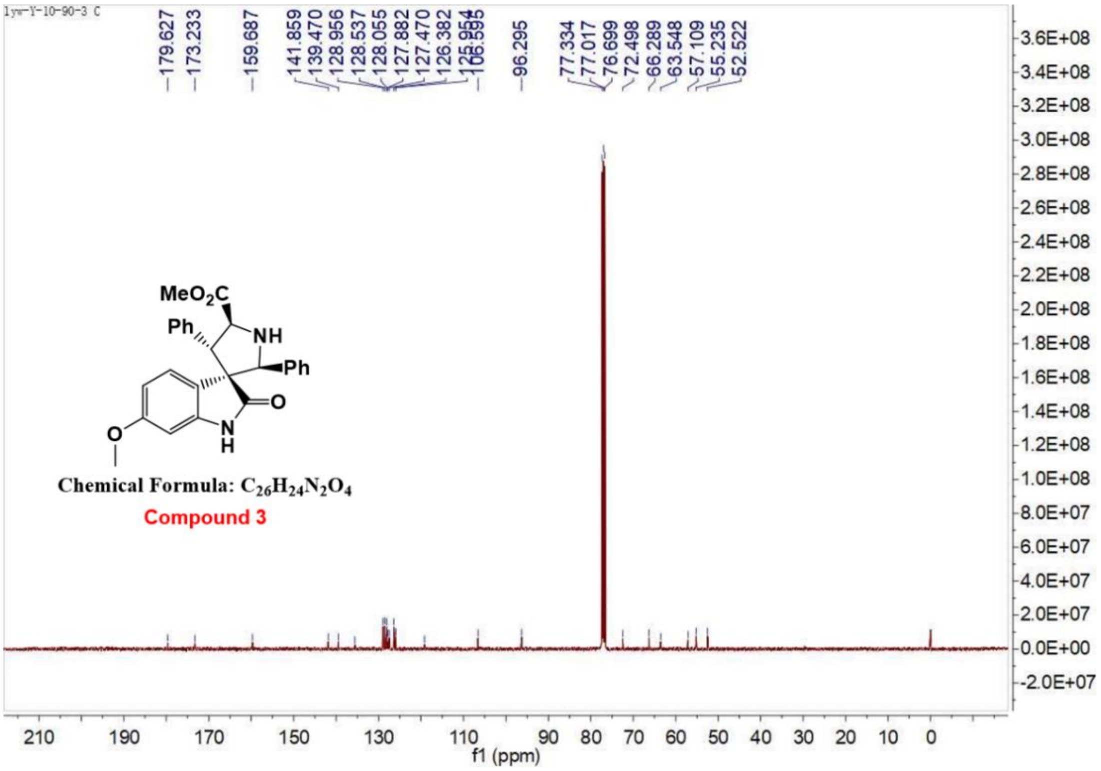

Figure S5. ^{13}C-NMR of **Compound 3**.

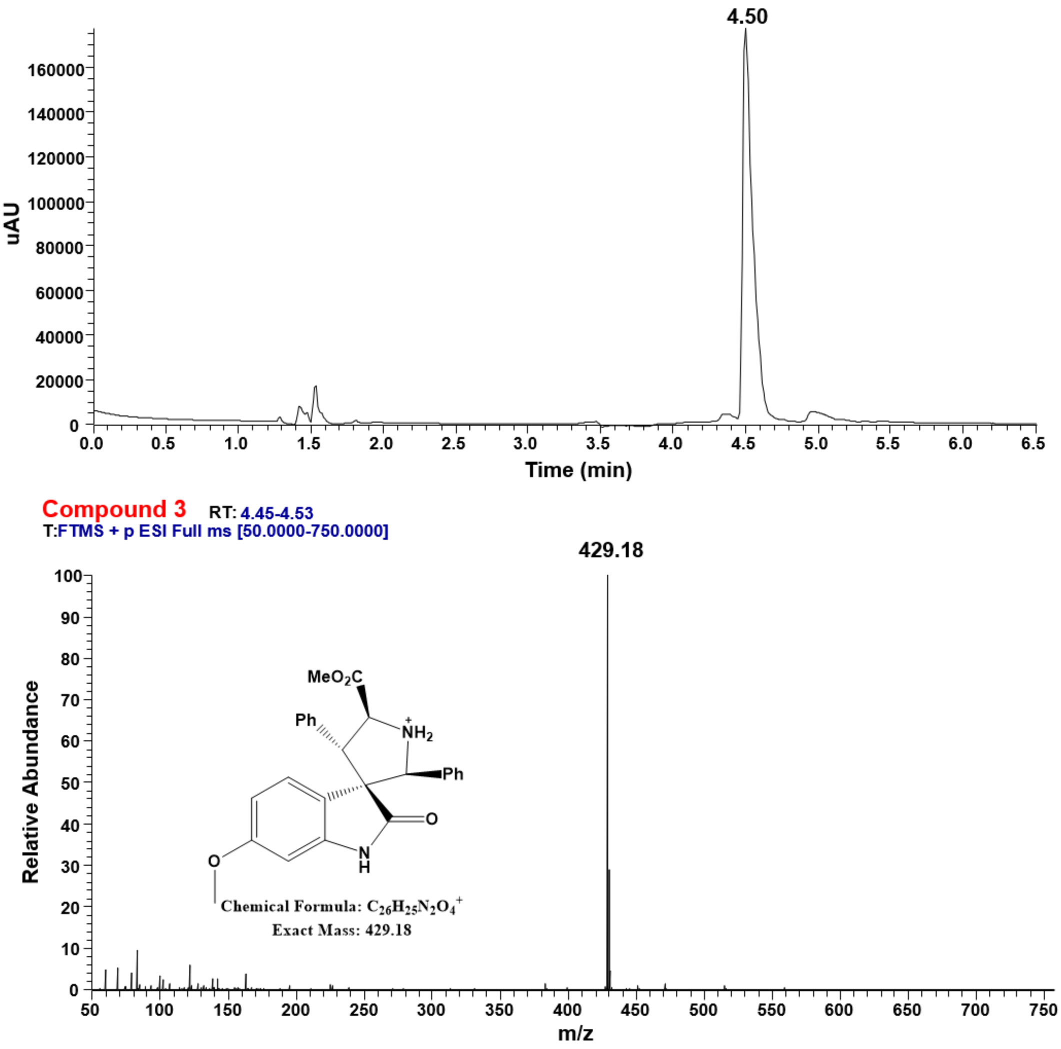

Figure S6. Mass spectra of **Compound 3**.

1.5 Molecular docking results of inhibitor-IDO1 complex

1.5.1 The overall structure of inhibitor-IDO1 complex

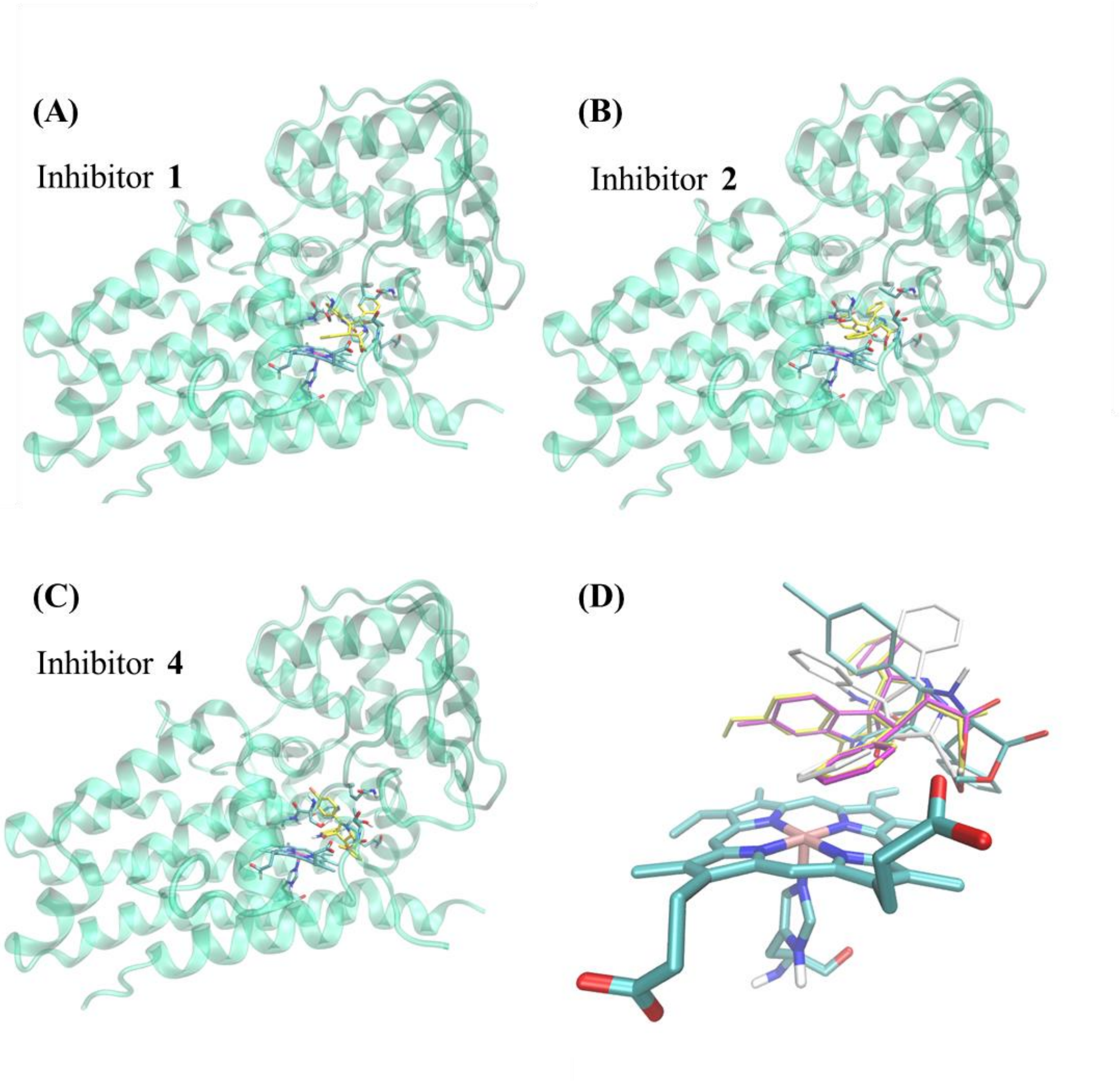

Figure S7. The overall structure of docking result of inhibitor **1 (A)**, inhibitor **2 (B)** inhibitor **4 (C)** binding to IDO1, respectively; **(D)** Structure of inhibitor-IDO1 active site. Heme is shown as stick model in cyan, Inhibitor **1**, **2**, **3**, **4** are shown as stick model in white, purple, yellow and cyan, respectively.

1.5.2 The docking energy of inhibitor-IDO1 complex

Table S1 Inhibitor 1-IDO1 complex docking results of AutoDock program

Model	$E_{binding}$ [a] (kcal/mol)	$E_{inter\text{-}mol}$ [b] (kcal/mol)	E_{vdw} [c] (kcal/mol)	E_{elec} [d] (kcal/mol)
1	-4.44	-5.64	-1.53	-0.16
2	-4.36	-5.56	-4.06	-0.16
3	-2.89	-4.09	0.0	-0.04
4	-2.81	-4.00	0.0	-0.04
5	-2.73	-3.92	0.0	-0.04
6	-2.73	-3.92	0.0	-0.04
7	-2.69	-3.89	0.0	-0.04
8	-2.67	-3.86	0.0	-0.04
9	-2.67	-3.87	0.0	-0.04
10	-2.66	-3.85	0.0	-0.04

[a] Binding energy. [b] Intermolecular energy. [c] Van der Waals energies. [d] Electrostatic interactions.

Table S2 Inhibitor 2-IDO1 complex docking results of AutoDock program

Model	$E_{binding}$ [a] (kcal/mol)	$E_{inter\text{-}mol}$ [b] (kcal/mol)	E_{vdw} [c] (kcal/mol)	E_{elec} [d] (kcal/mol)
1	-4.04	-5.53	-2.16	-0.21
2	-3.22	-4.71	-1.29	-0.45
3	-2.68	-4.17	0.0	-0.04
4	-2.64	-4.13	0.0	-0.04
5	-2.62	-4.11	0.0	-0.04
6	-2.57	-4.06	0.0	-0.04
7	-2.51	-4.00	0.0	-0.04
8	-2.48	-3.98	0.0	-0.04
9	-2.40	-3.89	0.0	-0.04
10	-2.38	-3.88	0.0	-0.04

[a] Binding energy. [b] Intermolecular energy. [c] Van der Waals energies. [d] Electrostatic interactions.

Table S3 Inhibitor 4-IDO1 complex docking results of AutoDock program

Model	$E_{binding}$ [a] (kcal/mol)	$E_{inter-mol}$ [b] (kcal/mol)	E_{vdw} [c] (kcal/mol)	E_{elec} [d] (kcal/mol)
1	-3.46	-4.65	-3.08	-0.11
2	-3.42	-4.61	-1.51	-0.05
3	-3.25	-4.44	-1.89	-0.10
4	-3.17	-4.36	-0.05	-0.08
5	-3.14	-4.33	-0.04	-0.08
6	-3.12	-4.32	-0.02	-0.09
7	-3.11	-4.30	-0.06	-0.08
8	-3.10	-4.29	-0.07	-0.08
9	-3.09	-4.28	-0.07	-0.10
10	-3.09	-4.28	-0.04	-0.08

[a] Binding energy. [b] Intermolecular energy. [c] Van der Waals energies. [d] Electrostatic interactions.

Reference

1. Zhang, J.X.; Wang, H.Y.; Jin, Q.W.; Zheng, C.W.; Zhao, G.; Shang, Y.J. Thiourea-Quaternary Ammonium Salt Catalyzed Asymmetric 1, 3-Dipolar Cycloaddition of Imino Esters To Construct Spiro[pyrrolidin-3,3'-oxindoles]. *Org. Lett.* **2016**, *18*, 4774-4777.

2. Wang G.; Liu X.; Huang T.; Kuang Y.; Lin L.; Feng X. Asymmetric catalytic 1,3-dipolar cycloaddition reaction of nitrile imines for the synthesis of chiral spiro-pyrazoline-oxindoles. *Org. Lett.* **2013**, *15*, 76-79.
